# Paroxysmal kinesigenic dyskinesia associated with a novel *POLG* variant: A case report

**DOI:** 10.1097/MD.0000000000024395

**Published:** 2021-01-29

**Authors:** Yaping Zhou, Jian Zhang, Xiaoting Wang, Qian Peng, Xiuli Shang

**Affiliations:** Department of Neurology, The First Hospital of China Medical University, Heping District, Shenyang, China.

**Keywords:** case report, movement disorder, paroxysmal kinesigenic dyskinesia, polymerase gamma, variant

## Abstract

**Introduction:**

Paroxysmal kinesigenic dyskinesia (PKD) is a rare neurological disease characterized by recurrent dyskinesia or choreoathetosis triggered by sudden movements. Pathogenic variants in *PRRT2* are the main cause of PKD. However, only about half of clinically diagnosed PKD patients have *PRRT2* mutations, indicating that additional undiscovered causative genes could be implicated. PKD associated with *POLG* variant has not been reported.

**Patient concerns:**

A 14-year-old boy presented with a 2-month history of involuntary dystonic movements triggered by sudden activities. He was conscious during the attacks. Neurological examination, laboratory tests, brain magnetic resonance imaging (MRI), electroencephalogram (EEG) were all normal. Genetic analysis showed a novel variant of *POLG* (c.440G>T, p.Ser147Ile), which was considered to be a likely pathogenic variant in this case.

**Diagnoses:**

The patient was diagnosed with PKD.

**Interventions:**

Low dose carbamazepine was used orally for treatment.

**Outcomes:**

The patient achieved complete resolution of symptoms without any dyskinesia during the 6-month follow up.

**Conclusion:**

Our study identified the novel *POLG* variant (c.440G>T, p.Ser147Ile) to be a likely pathogenic variant in PKD.

## 1 Introduction

Paroxysmal kinesigenic dyskinesia (PKD, OMIM #128200) is a rare neurological disease characterized by recurrent attacks of transient involuntary dystonia or choreoathetosis movements triggered by sudden activities.^[1,2]^ The prevalence of PKD is about 1 in 150,000, and the average time to obtain a correct diagnosis is almost 5 years due to lack of recognition.^[2,3]^ The commonly used diagnostic criteria of PKD was proposed by Bruno et al^[3]^ in 2004, which is based on history, clinical observation, imaging, and laboratory test results. Genetic advances led to greater diagnostic certainty. In 2011, researchers founded the *PRRT2* gene as a primary causative gene of PKD with an autosomal dominant inheritance pattern.^[4]^ However, only about 50% of primary PKD patients have *PRRT2* variants,^[5,6]^ suggesting that some other genes may be responsible for PKD. Therefore, finding new pathogenic genes and variants may provide an unequivocal diagnosis of the disease. In the current study, we described a case of clinically diagnosed PKD patient with a novel heterozygous *POLG* variant, which may broaden the genetic spectrum of PKD.

## 2 Case report

A 14-year-old boy presented with a 2-month history of involuntary dystonic movements triggered by sudden activities after a period of physical rests. The involuntary movements last approximately 10 seconds. Attack frequency varies from about 20 times per day to once for a couple of weeks. Both sides of his body could be involved, accompanied by occasional spasmodic torticollis. Stress and anxiety can increase the likelihood of episodes. He was unable to control the attacks. His consciousness was unaffected during the process. He was previously healthy without known significant abnormalities during his birth and growth. His family history was not notable for involuntary movements, epilepsy, or other related diseases.

On physical examination, the vital signs were unremarkable and neurological examination was normal.

**Figure 1:**
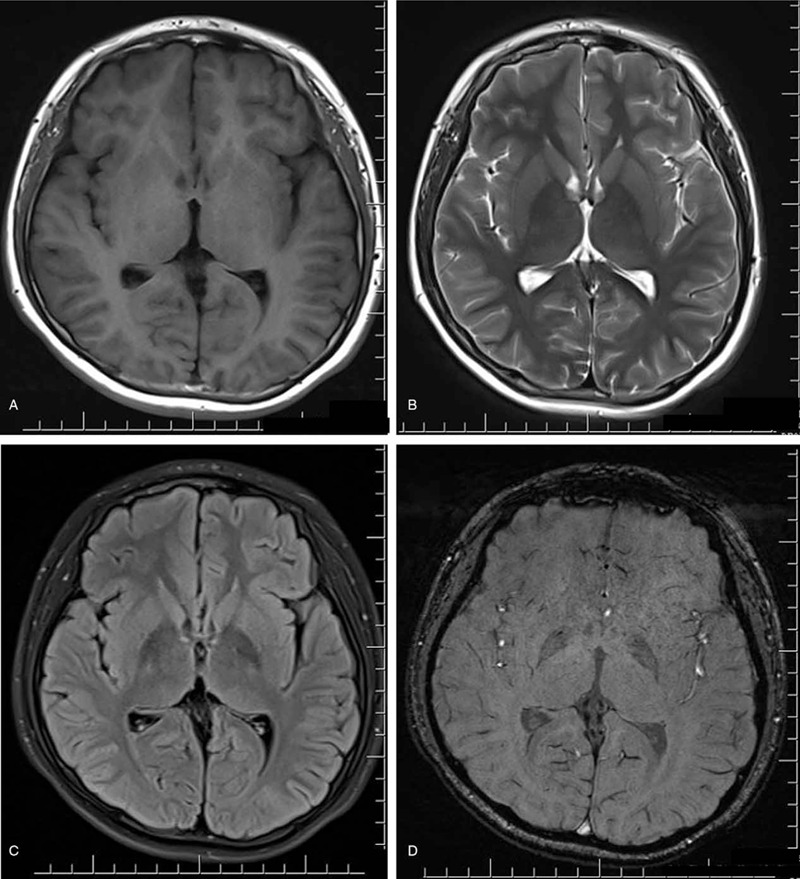
Brain MRI: (A) T1-weighted, (B) T2-weighted, and (C) fluid-attenuated inversion recovery (FLAIR) imaging were all normal, with no intracranial space-occupying lesion, parenchyma atrophy, or other abnormal changes; (D) susceptibility weighted imaging (SWI) showing no abnormal iron deposition. MRI = magnetic resonance imaging.

Laboratory test results were within normal limits, including routine serum tests, standard biochemistry profile, serum lactate concentration, ceruloplasmin, etc. Brain magnetic resonance imaging (MRI) was normal (Fig. 1). Electroencephalogram (EEG) was normal.

**Figure 2:**
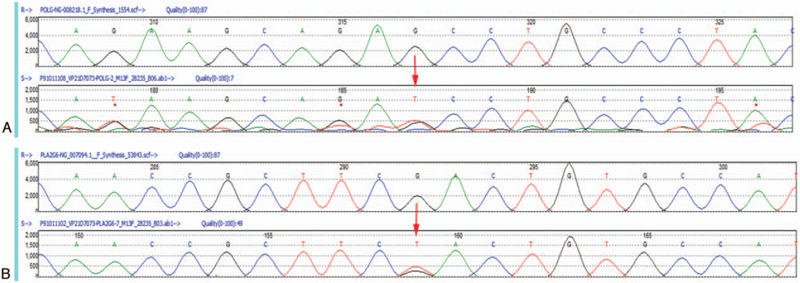
The electropherogram from the proband. (A) Identification of a novel heterozygous missense *POLG* variant (c.440G>T, p.Ser147Ile) in exon 2. (B) Identification of a heterozygous *PLA2G6* variant (c.991G>T, p.Asp331Tyr) in exon 7. The arrows indicate the positions of transition.

A diagnosis of PKD was concluded, and genetic analysis was carried out with a written informed consent obtained from his parents. High-throughput sequencing and Sanger sequencing were performed. Heterozygous variants were found in *POLG* (NM_002693.2, Exon2, c.440G>T, p.Ser147Ile) and *PLA2G6* (NM_003560.3, Exon7, c.991G>T, p.Asp331Tyr) (Fig. 2). Both mutant alleles were inherited from his asymptomatic mother. The *POLG* variant (c.440G>T, p.Ser147Ile) located on the exonuclease domain, one of the important functional domains of POLG1 protein, and may lead to infidelity of mitochondrial DNA (mtDNA) replication and proofreading errors.^[7,8]^ This variant was novel and not registered in the following genetic public database: Human Gene Mutation Database (HGMD), ESP6500, 1000 Genomes Project, ClinVar, and dbSNP. The deleterious effect was predicted by multiple programs in silico, including PolyPhen-2, SIFT, MutationTaster, MutationAssessor, FATHMM, GERP, PhyloP, and SiPhy. The *POLG* variant was thus assigned as likely pathogenic according to the guidelines of the American College of Medical Genetics and Genomics.^[9]^ The missense *PLA2G6* variant (c.991G>T, p.Asp331Tyr) has been reported in Parkinsonism patients with an autosomal recessive inheritance pattern.^[10]^ Thus, the *PLA2G6* variant was considered less likely to be the causal gene in this case.

Oral carbamazepine (CBZ) 200 mg/d was used for treatment, and the dosages gradually reduced to 50 mg/d within 2 weeks. He achieved complete resolution of symptoms within 24 hours after he took medicine and reported no involuntary movements attacks when the dosage decreased to 50 mg/d. During the 6-month follow up, the PKD episodes vanished entirely without a single attack since medicine use.

This study was approved by the Ethics Committee of the First Hospital of China Medical University and adhered to the tenets of the Declaration of Helsinki.

## 3 Discussion

The present study described a clinically diagnosed PKD patient harboring heterozygous variants in *POLG* and *PLA2G6*. No variant in *PRRT2* was detected. The heterozygous *POLG* variant was novel and considered to be likely pathogenic in this case according to the guidelines of the American College of Medical Genetics and Genomics.^[9]^ This patient meets the criteria for PKD as proposed by Bruno et al,^[3]^ including the onset of symptoms at 14 years old, identified kinesigenic trigger for attacks, short duration of attacks (<1 minute), unaffected consciousness during attacks, and without other organic disease or abnormal neurological examination. Moreover, low does CBZ could resolve the PKD episodes entirely, which was in accordance with previous studies.^[11]^ PKD patients can manifest choreoathetosis movements, dystonic movements, or the mixed type, and most patients primarily present with dystonic movements.^[3,12]^ Our case mainly presented with dystonic movements.

The *POLG* gene, located on chromosome 15q25, encodes polymerase gamma 1 (POLG1), which is an enzyme responsible for the repair and replication of mtDNA (16). The POLG1 enzyme is comprised of 3 main functional domains, including exonuclease domain (amino acid residues 26-417), linker domain (amino acid residues 418–755), and polymerase domain (amino acid residues 756–1239).^[8,13–15]^ In our study, the variant (c.440G>T, p.Ser147Ile) located on the exonuclease domain and should lead to the infidelity of mtDNA replication and errors in proofreading.^[7,8]^ The *POLG*-related disease is of broad-spectrum and significant heterogeneity, including progressive external ophthalmoplegia (PEO),^[16]^ autosomal recessive and dominant PEO,^[16,17]^ sensory ataxic neuropathy, dysarthria and ophthalmoparesis,^[18]^ autosomal recessive ataxia,^[19,20]^ spinocerebellar ataxia with epilepsy,^[21]^ etc. Individual presentations vary a lot and are influenced by multiple factors, including *POLG* genotype, genetic background, epigenetic effects, environmental factors and the age of onset.^[8]^ Dystonia and movement disorders are common presentations in *POLG*-related diseases.^[22,23]^

The pathophysiological mechanisms of *POLG* variant in PKD are unclear. Although multiple studies have been carried out on PKD, knowledge about the pathogenic mechanisms is limited.^[24]^ Although the channelopathy hypothesis is prevailing in PKD, it is insufficient to explain the pathophysiology fully.^[25]^ A recent study reported a complicated case of PKD with *SACS* mutation.^[26]^ The *SACS* gene encodes mitochondrial protein sacsin, and variants of *SACS* result in defects in mitochondrial dynamics. This study indicated that mitochondria might play a role in the pathophysiology of PKD. Our report could be the second research about PKD associated with genes that may affect mitochondrial function. The identification of additional PKD cases associated with *POLG* variants and further functional studies are warranted.

The association between mitochondrial disorders and paroxysmal dyskinesias have also been reported in several other studies. Mutations in *ECHS1* (enoyl CoA hydratase, short chain, 1, mitochondrial), encoding for the short-chain enoyl-CoA hydratase protein (SCEH), have been reported as a novel cause of paroxysmal exercise-induced dyskinesia (PED).^[27,28]^ Paroxysmal non-kinesigenic dyskinesia (PNKD) can also occur in patients carrying variants in BCKD complex, which functioned as mitochondrial branched chain alpha ketoacid dehydrogenase kinase.^[25]^

Although most of the *POLG*-related disorders are autosomal dominant or autosomal recessive, variably penetrant and incompletely penetrant dominant variants have also been reported.^[8,18,29–31]^ Burusnukul and de los Reyes^[29]^ has reported a case of 2 half-siblings with heterozygous *POLG* variant (p.Gly517Val). The variant site located in the linker region between the exonuclease and polymerase domains of POLG1. The patients showed multiple symptoms, including early-onset seizures, myoclonus, hypotonia, and developmental delay, but their carrier mother was unaffected,^[29]^ much as was the case in our study. Unfortunately, since limited family members of the patient were available in genetic analysis in our research, the exact inheritance pattern was unclear at this stage. These limitations also existed in previous similar studies.^[26–28]^ Further clinical and biological researches are needed.

## 4 Conclusion

In conclusion, our study suggests a novel heterozygous *POLG* variant in a patient with PKD, which may expend the gene spectrum of PKD and help to diagnose precisely in PKD patients without *PRRT2* mutations. The mitochondrial pathway may be a possible pathophysiology mechanism of PKD, and further functional analyses are needed.

## Acknowledgments

The authors thank the Shenyang KingMed for Clinical Laboratory for the assistance in gene sequencing, as well as the patient and his parents for their participation in this study.

## Author contributions

**Formal analysis:** Yaping Zhou.

**Funding acquisition:** Xiuli Shang.

**Investigation:** Yaping Zhou.

**Project administration:** Xiuli Shang.

**Resources:** Xiuli Shang.

**Supervision:** Xiuli Shang.

**Validation:** Xiuli Shang.

**Visualization:** Xiuli Shang.

**Writing – original draft:** Yaping Zhou.

**Writing – review & editing:** Yaping Zhou, Jian Zhang, Xiaoting Wang, Qian Peng, Xiuli Shang.

## Abbreviations

Abbreviations: CBZ = carbamazepine, ECHS1 = enoyl CoA hydratase, short chain, 1, EEG = electroencephalogram, MRI = magnetic resonance imaging, mtDNA = mitochondrial DNA, PED = paroxysmal exercise-induced dyskinesias, PEO = progressive external ophthalmoplegia, PKD = paroxysmal kinesigenic dyskinesia, PNKD = paroxysmal non-kinesigenic dyskinesia, POLG1 = polymerase gamma 1, PRRT2 = proline-rich transmembrane protein 2, SCEH = short-chain enoyl-CoA hydratase protein.
